# Impact of interpregnancy interval after pregnancy loss on clinical pregnancy and neonatal outcomes of subsequent frozen-thawed embryo transfer cycles: a retrospective cohort study

**DOI:** 10.1186/s12958-026-01556-7

**Published:** 2026-04-22

**Authors:** Wanli Yang, Shasha Wang, Qianqian Zhu, Mingru Yin, Pengcheng Kong

**Affiliations:** 1 Center of Reproductive Medicine, Shanghai First Maternity and Infant Hospital, School of Medicine, Tongji University, 2699 gao-ke-xi Rd, Shanghai, 201204 China; 2 Department of Psychiatry and Psychology, Shanghai Children’s Hospital, School of medicine, Shanghai Jiao Tong University, Shanghai, China; 3 Department of Assisted Reproduction, Shanghai Ninth People’s Hospital, Jiao Tong University School of Medicine, 639 Zhi-zao-ju Rd, Shanghai, 200011 China

**Keywords:** Interpregnancy interval, Pregnancy loss, Frozen–thawed embryo transfer, Pregnancy outcomes, Neonatal outcomes

## Abstract

**Background:**

The optimal interpregnancy interval (IPI) before reinitiating frozen-thawed embryo transfer (FET) after pregnancy loss remains uncertain, particularly in women undergoing assisted reproductive technology. Current guidelines recommending a waiting period are largely based on spontaneous conception data and may not apply to FET cycles. This study aimed to evaluate whether the length of IPI following biochemical or clinical pregnancy loss affects subsequent reproductive and neonatal outcomes in an infertile FET population.

**Methods:**

In this retrospective cohort study, 2,620 infertile women who experienced a biochemical or clinical pregnancy loss after a preceding FET cycle and subsequently underwent a consecutive FET cycle derived from the same oocyte retrieval at a tertiary academic center between January 2011 and December 2022 were included. IPI was defined as the interval from the end of the failed pregnancy to the subsequent embryo transfer and categorized as < 6 months, 6–12 months, or 12–24 months. Live birth was the primary outcome. Secondary outcomes included conception, clinical pregnancy, pregnancy loss, and adverse neonatal outcomes. Multivariable logistic regression models were used to estimate adjusted odds ratios (aORs) with 95% confidence intervals (CIs).

**Results:**

Using the IPI of 6–12 months as the reference, a shorter IPI (< 6 months) was not associated with a reduced likelihood of clinical pregnancy among women with prior biochemical pregnancy loss (aOR 1.29; 95% CI 0.88–1.89) or clinical pregnancy loss (aOR 0.93; 95% CI 0.71–1.21). Similarly, the odds of live birth were comparable between women who reinitiated FET within 6 months and those who waited 6–12 months, regardless of the type of preceding pregnancy loss (biochemical pregnancy loss: aOR 1.18; 95% CI 0.80–1.74; clinical pregnancy loss: aOR 0.94; 95% CI 0.73–1.23). Extending the IPI to 12–24 months did not confer additional benefits for reproductive outcomes. Rates of adverse neonatal outcomes, including preterm birth, low birth weight, and small for gestational age, were similar across all IPI categories.

**Conclusions:**

Among infertile women undergoing FET after a biochemical or clinical pregnancy loss, reinitiating FET within 6 months was not associated with compromised reproductive or neonatal outcomes. These findings support the safety of early FET reinitiation and suggest that delaying subsequent treatment in the absence of medical indications may be unnecessary.

**Supplementary Information:**

The online version contains supplementary material available at 10.1186/s12958-026-01556-7.

## Background

Pregnancy loss affects approximately 12%–15% of clinically recognized pregnancies, imposing substantial physical and emotional burdens on affected women [1–3]. For infertile women undergoing assisted reproductive technology (ART), the pressure to conceive is often intensified by advancing maternal age and declining ovarian reserve [4]. Consequently, determining the optimal IPI following pregnancy loss represents a critical clinical challenge that requires balancing adequate physiological recovery against the constraints of the reproductive biological clock.

Existing evidence regarding the optimal IPI after pregnancy loss remains inconclusive. While several studies have reported no increased risk of adverse obstetric or perinatal outcomes associated with shorter waiting times before the subsequent pregnancy [5, 6], others have suggested that a shorter IPI may be linked to unfavorable outcomes, potentially due to inadequate physiological or psychological recovery following the preceding loss [7–9]. In clinical practice, many physicians recommend waiting at least three months after a miscarriage to reduce the risk of a subsequent loss [10, 11]. However, this recommendation is largely derived from observational studies in naturally conceiving populations, in which interpretation is complicated by fecundity-related confounding, as shorter IPIs may preferentially identify women with inherently higher fertility.

Whether these observations can be extrapolated to women undergoing ART remains uncertain. Unlike spontaneous conception, ART cycles are clinically scheduled, and in FET cycles, embryos are typically generated from oocytes retrieved during a prior stimulation cycle, thereby minimizing confounding related to progressive oocyte aging over time. Moreover, the physiological and endocrine recovery following an ART-related biochemical or clinical pregnancy loss may differ from that observed after spontaneous conception, raising the possibility that the optimal IPI in this setting may not mirror that of natural conception. A recent large cohort study by Wang et al. reported poorer outcomes associated with shorter IPIs among women undergoing frozen blastocyst transfer [7]. However, the restriction to blastocyst-stage transfers limits the generalizability of these findings, as cleavage-stage embryo transfer remains widely practiced in routine clinical settings. In addition, accumulating evidence suggests that blastocyst transfer itself may be associated with an increased risk of adverse perinatal outcomes, including preterm birth, large-for-gestational-age infants, and congenital anomalies [12, 13].

Given the continued use of cleavage-stage embryo transfer and the lack of consensus regarding the optimal timing of subsequent FET following pregnancy loss, robust, ART-specific evidence is urgently needed to inform individualized clinical counseling. Therefore, this retrospective cohort study aimed to evaluate the association between IPI length after biochemical or clinical pregnancy loss and subsequent reproductive, obstetric, and neonatal outcomes, and to explore whether these associations differ according to embryo developmental stage.

## Materials and methods

### Study design and population

This study was approved by the Ethics Committee (Institutional Review Board) of the Shanghai Ninth People’s Hospital (approval No. SH9H-2023-T213-1). The requirement for informed consent was waived by the Ethics Committee due to the retrospective nature of the study and the use of anonymized data. The study was conducted in accordance with the Strengthening the Reporting of Observational Studies in Epidemiology (STROBE) guidelines.

Clinical data were extracted from the electronic database of the Assisted Reproduction Center at Shanghai Ninth People’s Hospital. In accordance with the Technical Standards for Human Assisted Reproduction issued by the Chinese Ministry of Health, detailed information on ART treatment characteristics and neonatal outcomes has been prospectively recorded in this database. Data accuracy and completeness were ensured through standardized data entry, routine cross-checking by trained physicians, and independent verification by dedicated data management personnel.

**Fig. 1:**
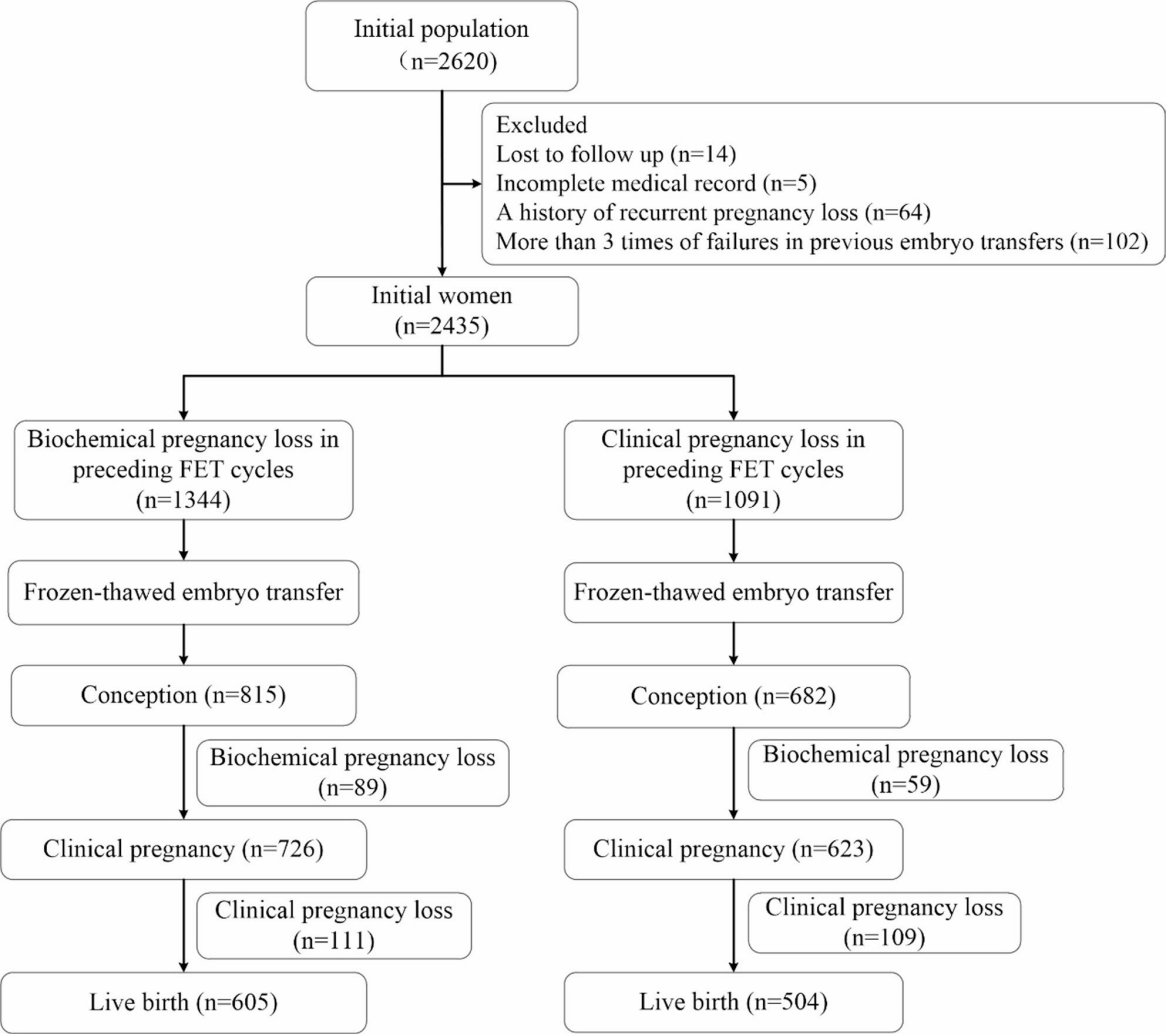
Flowchart of the study population

This study included infertile women who experienced pregnancy loss following a FET and subsequently underwent another FET between January 1, 2011 and December 31, 2022 (Fig. 1). In this study, pregnancy loss was strictly defined. Biochemical pregnancy loss was defined as a transient elevation in serum human chorionic gonadotropin (hCG) without ultrasonographic evidence of a gestational sac. Clinical pregnancy loss was defined as the loss of a clinically confirmed intrauterine pregnancy before 24 completed weeks of gestation. Selective terminations following abnormal prenatal screening or diagnostic results were specifically excluded, as their etiology differs fundamentally from that of spontaneous pregnancy loss. Only women whose two consecutive FET cycles involved embryos derived from the same oocyte retrieval cycle were eligible for inclusion, thereby minimizing confounding related to oocyte aging or stimulation variability. Embryo quality was assessed according to the Gardner grading system [14]. Furthermore, the subsequent FET cycle was initiated only when the endometrial thickness reached our institutional minimum threshold (≥ 7 mm) with satisfactory morphology, thereby ensuring adequate physiological recovery prior to transfer. Each patient contributed only one pair of consecutive cycles (the loss cycle and the subsequent FET cycle) to the analysis.

Women with recurrent pregnancy loss (defined as two or more consecutive spontaneous clinical pregnancy losses, *n* = 64), those who failed to conceive after more than three embryo transfer cycles (*n* = 102), women lost to follow-up (*n* = 14), and those with incomplete medical records (*n* = 5) were excluded. Additional exclusion criteria included uterine anatomical abnormalities, oocyte donation cycles, ectopic pregnancy in the preceding cycle, late pregnancy loss (≥ 24 weeks of gestation) or stillbirth in the preceding cycle, and preimplantation genetic testing (PGT) cycles. The final study population consisted of 2,620 women, including 1,344 women with a preceding biochemical pregnancy loss and 1,091 women with a preceding clinical pregnancy loss, all of whom proceeded to a subsequent FET cycle.

### Definition of interpregnancy interval and institutional guidelines

The IPI is conventionally defined as the time between the end of a pregnancy and the conception of the subsequent pregnancy. In the context of ART, IPI was operationalized as the interval between the date of pregnancy loss following the preceding FET and the initiation of the subsequent FET cycle, reflecting a clinically actionable treatment interval. Specifically, IPI was calculated as the number of days from the documented date of pregnancy loss to the first day of the subsequent FET cycle. For women with biochemical pregnancy loss, IPI was calculated from the date when serum hCG declined to < 5 IU/L to the first day of the subsequent FET cycle. IPI was categorized into three groups: < 6 months, 6–12 months, and 12–24 months, with the 6–12 month group serving as the reference category. In our institution, clinical guidelines also influence the timing of FET resumption. For biochemical pregnancy loss, patients are generally advised to wait for one or two normal menstrual cycles (approximately 1–2 months) to allow for complete endometrial shedding and hormonal normalization. For clinical pregnancy loss, especially if surgical management (e.g., dilation and curettage) is required, a minimum waiting period of 2–3 months is typically recommended to facilitate complete endometrial repair.

### Outcome measures

The primary outcome was live birth following the subsequent FET cycle. Consistent with our previously published work, live birth was defined as the delivery of at least one infant at ≥ 24 weeks of gestation who survived beyond 28 days after birth [15]. Secondary outcomes included pregnancy outcomes and adverse neonatal outcomes. Pregnancy outcomes encompassed conception (defined as serum hCG ≥ 5 IU/L 14 days after FET), clinical pregnancy (confirmed by transvaginal ultrasonography demonstrating ≥ 1 gestational sac at 30–35 days after FET), biochemical pregnancy loss, and clinical pregnancy loss in the subsequent cycle. Adverse neonatal outcomes included preterm birth (delivery before 37 completed weeks of gestation), low birth weight (LBW, defined as birth weight < 2,500 g), and small for gestational age (SGA, defined as birth weight below the 10th percentile for gestational age and sex according to Chinese reference data [16].

### Statistical analysis

Baseline demographic characteristics and cycle parameters were summarized according to IPI categories (< 6 months, 6–12 months, and 12–24 months). Continuous variables were presented as mean ± standard deviation (SD) and compared using the Student’s ttest or analysis of variance (ANOVA), while categorical variables were expressed as frequencies (percentages) and compared using the Chi-square test or Fisher’s exact test, as appropriate.

The statistical analysis plan, including the predefined primary and secondary outcomes and the selection of covariates, was established a priori before data extraction. Multivariable logistic regression models were constructed to estimate odds ratios (ORs) and 95% confidence intervals (CIs) for the associations between IPI and reproductive and neonatal outcomes. Covariates included in the adjusted models were selected based on statistical significance in univariable analyses and biological plausibility, including maternal age, body mass index, duration of infertility, gravidity, parity, infertility diagnosis (tubal factor, polycystic ovary syndrome, endometriosis, diminished ovarian reserve, male factor, or other causes), number of transferred embryos, embryo developmental stage, embryo quality, endometrial preparation protocol (natural cycle, hormone replacement cycle, or stimulated cycle), and endometrial thickness.

Several sensitivity and subgroup analyses were performed. First, sensitivity analyses were restricted to women aged < 35 years to minimize confounding by maternal age. Second, sensitivity analyses were limited to cycles involving cleavage-stage embryo transfer to assess the potential influence of embryo developmental stage. Third, sensitivity analyses were restricted to cycles with good-quality embryos to evaluate the robustness of the findings with respect to embryo quality. Finally, predefined subgroup analyses were conducted by stratifying the clinical pregnancy loss cohort according to the gestational age at loss and the abortion method (medical vs. surgical) to explore potential heterogeneity.

All statistical analyses were conducted using Stata software (version 14.0; StataCorp, College Station, TX, USA). A two-sided P value < 0.05 was considered statistically significant.

## Results

A total of 2,620 infertile women were enrolled in this study. Among them, 1,344 women experienced a preceding biochemical pregnancy loss, and 1,091 women experienced a preceding clinical pregnancy loss, all of whom subsequently underwent FET (Fig. 1).

Baseline demographic characteristics and cycle parameters of the study cohort across the three IPI groups are summarized in Table 1. Among the 1,344 women with biochemical pregnancy loss in the preceding FET cycle, 1,182 (87.95%) initiated the subsequent FET within 6 months, 126 (9.38%) between 6 and 12 months, and 36 (2.68%) between 12 and 24 months. Of the 1,091 women with prior clinical pregnancy loss, 490 (44.91%) restarted FET treatment within 6 months, 454 (41.61%) after 6–12 months, and 147 (13.47%) after 12–24 months. Due to institutional guidelines requiring adequate physical recovery, no patients underwent FET within 1 month. The minimum IPI observed was 42 days for the biochemical loss group and 72 days for the clinical loss group. Importantly, endometrial thickness was highly comparable and showed no significant differences across the IPI groups (10.58 ± 2.28 vs. 10.31 ± 2.31 vs. 10.40 ± 2.61 mm, *P* = 0.066), indicating standardized endometrial preparation prior to transfer. When comparing baseline characteristics across the three IPI groups, patients with a longer IPI were significantly older (32.68 ± 4.63 vs. 33.44 ± 4.54 vs. 34.50 ± 4.54 years, *P* < 0.001). Tubal factor infertility was the most common diagnosis across all groups. Stimulated cycles were less frequently used as the IPI increased (49.52% vs. 42.24% vs. 41.53%, *P* = 0.004), and women with a shorter IPI were more likely to receive a transfer of two or more embryos (80.20% vs. 69.83% vs. 61.20%, *P* < 0.001). Overall, approximately 77% to 80% of cycles involved cleavage-stage embryo transfer, and more than 85% of transferred embryos were of good quality, with the majority of women receiving transfer of two or more embryos.

**Table 1:** Baseline demographics and cycle characteristics of the study cohort

Characteristic	Interpregnancy intervals (months)			*P* value
	< 6	6–12	12–24	
Number of women, n	1672	580	183	
Maternal age, mean (SD), y	32.68 (4.63)	33.44 (4.54)	34.50(4.54)	< 0.001
Body mass index, mean (SD), kg/m^2^	21.99(5.61)	22.29 (3.96)	22.73(3.91)	0.122
Infertility duration, mean (SD), y	3.23(2.93)	3.33 (2.94)	3.38(2.81)	0.673
Gravidity				
0	890(53.23)	296(51.03)	92(50.27)	0.262
1	394(23.56)	162(27.93)	45(24.59)	
≥2	388(23.21)	122(21.03)	46(25.14)	
Parity				
0	1463(87.50)	513(88.45)	166(90.71)	0.412
≥ 1	209(12.50)	67(11.55)	17(9.29)	
Cause of infertility				0.335
Tubal factor	783(46.83)	243(41.90)	96(52.46)	
Polycystic ovarian syndrome	203(12.14)	82(14.14)	23(12.57)	
Diminished ovarian reserve	41(2.45)	17(2.93)	5(2.73)	
Endometriosis	194(11.60)	78(13.45)	17(9.29)	
Male factor	366(21.89)	129(22.24)	30(16.39)	
Other factors	85(5.08)	31(5.34)	12(6.56)	
Endometrial preparation				
Natural cycles	302(18.06)	139(23.97)	41(22.40)	0.004
Hormone replacement cycles	542(32.42)	196(33.79)	66(36.07)	
Stimulated cycles	828(49.52)	245(42.24)	76(41.53)	
Number of transferred embryos				
1	331(19.80)	175(30.17)	71(38.80)	< 0.001
≥2	1341(80.20)	405(69.83)	112(61.20)	
Endometrial thickness, mean (SD), mm	10.58(2.28)	10.31(2.31)	10.40(2.61)	0.066
Developmental stage of transferred embryos				
Cleavage-stage embryo	1287(76.97)	453(78.10)	146(79.78)	0.596
Blastocyst	373(22.31)	125(21.55)	37(20.22)	
Cleavage-stage embryo+blastocyst	12(0.72)	2(0.34)	0(0.00)	
Embryo quality				
Good-quality	1465(87.62)	517(89.14)	167(91.26)	0.529
Good-quality + poor-quality	108(6.46)	30(5.17)	9(4.92)	
Poor quality	99(5.92)	33(5.69)	7(3.83)	

As summarized in Table 2, we compared the crude pregnancy and neonatal outcomes in subsequent FET cycles across the three IPI categories for women with a prior biochemical or clinical pregnancy loss. The analysis revealed no significant inter-group differences in the rates of conception, clinical pregnancy, biochemical pregnancy loss, clinical pregnancy loss, live birth, or singleton live birth. Moreover, there were no significant differences in the rates of LBW and SGA across the three IPI groups, irrespective of the type of preceding pregnancy loss.

**Table 2:** Pregnancy and neonatal outcomes in subsequent FET cycles following prior pregnancy loss by interpregnancy interval

		Interpregnancy intervals (months)			*P* value
		< 6	6–12	12–24	
Patients with biochemical pregnancy loss in preceding FET cycles	Conception	727(61.51)	65(51.59)	23(63.89)	0.088
	Clinical pregnancy	646(54.65)	59(46.83)	21(58.33)	0.214
	Biochemical pregnancy loss	81(6.85)	6(4.76)	2(5.56)	0.695
	Clinical pregnancy loss	100(8.46)	8(6.35)	3(8.33)	0.765
	Live birth	538(45.52)	50(39.68)	17(47.22)	0.441
	Live birth in singletons	393(33.25)	34(26.98)	16(44.44)	0.183
	Preterm birth in singletons	17(4.33)	2(5.88)	1(6.25)	0.585
	LBW in singletons	8(2.04)	2(5.88)	0	0.314
	SGA in singletons	18(4.58)	1(2.94)	0	1.000
Patients with clinical pregnancy loss in preceding FET cycles	Conception	307(62.65)	290(63.88)	85(57.82)	0.418
	Clinical pregnancy	278(56.73)	266(58.59)	79(53.74)	0.573
	Biochemical pregnancy loss	29(5.92)	24(5.29)	6(4.08)	0.681
	Clinical pregnancy loss	53(10.82)	48(10.57)	8(5.44)	0.141
	Live birth	223(45.51)	212(46.70)	69(46.94)	0.918
	Live birth in singletons	162(33.06)	158(34.80)	59(40.14)	0.289
	Preterm birth in singletons	14(8.64)	17(10.76)	4(6.78)	0.680
	LBW in singletons	7(4.32)	10(6.33)	2(3.39)	0.661
	SGA in singletons	4(2.47)	6(3.80)	4(6.78)	0.326

*Abbreviations*: *LBW* Low birth weight, *SGA* Small for gestational age

In multivariable logistic regression analyses adjusting for potential confounders (Table 3), no significant associations were observed between a short IPI (< 6 months) and the primary outcome of live birth when compared with the 6–12 months reference group. This held true for both women with prior biochemical pregnancy loss (aOR = 1.18; 95% CI, 0.80–1.74) and those with prior clinical pregnancy loss (aOR = 0.94; 95% CI, 0.73–1.23). Extending the IPI to 12–24 months was also not associated with improvements in live birth rates in either group. Among women with prior biochemical pregnancy loss, although a higher likelihood of conception was observed for those with an IPI of < 6 months in univariable analysis (OR = 1.50; 95% CI, 1.04–2.17), this association was no longer statistically significant after multivariable adjustment (aOR = 1.42; 95% CI, 0.97–2.07). Similarly, a shorter IPI (< 6 months) was not significantly associated with conception among women with preceding clinical pregnancy loss (aOR = 0.94; 95% CI, 0.71–1.23). Compared with an IPI of 6–12 months, a shorter IPI was not associated with clinical pregnancy in women with prior biochemical pregnancy loss (aOR = 1.29; 95% CI, 0.88–1.89) or clinical pregnancy loss (aOR = 0.93; 95% CI, 0.71–1.21). Furthermore, the adjusted odds of biochemical pregnancy loss and clinical pregnancy loss following subsequent FET did not differ significantly between women with an IPI of < 6 months and those with an IPI of 6–12 months.

**Table 3:** Crude and adjusted odds ratios for pregnancy and neonatal outcomes in subsequent FET cycles following prior pregnancy loss by interpregnancy interval

	Patients with biochemical pregnancy loss in preceding FET cycles		Patients with clinical pregnancy loss in preceding FET cycles	
	Crude OR (95%CI)	Adjusted OR (95%CI)	Crude OR (95%CI)	Adjusted OR (95%CI)
Conception				
IPI < 6 mon	1.50(1.04–2.17)	1.42(0.97–2.07)	0.95(0.73–1.24)	0.94(0.71–1.23)
IPI 6–12 mon	Ref	Ref	Ref	Ref
IPI 12–24 mon	1.66(0.77–3.57)	1.93(0.87–4.27)	0.78(0.53–1.13)	0.89(0.60–1.32)
Clinical pregnancy				
IPI < 6 mon	1.37(0.95–1.98)	1.29(0.88–1.89)	0.93(0.72–1.20)	0.93(0.71–1.21)
IPI 6–12 mon	Ref	Ref	Ref	Ref
IPI 12–24 mon	1.59(0.75–3.36)	1.85(0.85–4.01)	0.82(0.56–1.19)	0.94(0.63–1.39)
Biochemical pregnancy loss				
IPI < 6 mon	1.47(0.63–3.44)	1.47(0.62–3.49)	1.13(0.65–1.97)	1.05(0.59–1.85)
IPI 6–12 mon	Ref	Ref	Ref	Ref
IPI 12–24 mon	1.18(0.23–6.10)	1.19(0.23–6.28)	0.76(0.31–1.90)	0.77(0.30–1.98)
Clinical pregnancy loss				
IPI < 6 mon	1.36(0.65–2.87)	1.46(0.67–3.17)	1.03(0.68–1.55)	1.03(0.68–1.57)
IPI 6–12 mon	Ref	Ref	Ref	Ref
IPI 12–24 mon	1.34(0.34–5.34)	1.34(0.33–5.47)	0.49(0.22–1.05)	0.52(0.24–1.15)
Live birth				
IPI < 6 mon	1.27(0.87–1.85)	1.18(0.80–1.74)	0.95(0.74–1.23)	0.94(0.73–1.23)
IPI 6–12 mon	Ref	Ref	Ref	Ref
IPI 12–24 mon	1.36(0.65–2.87)	1.58(0.73–3.42)	1.01(0.70–1.46)	1.13(0.77–1.67)
Preterm birth in singletons				
IPI < 6 mon	0.69(0.23–2.06)	0.76(0.23–2.50)	0.72(0.37–1.39)	0.71(0.35–1.43)
IPI 6–12 mon	Ref	Ref	Ref	Ref
IPI 12–24 mon	1.08(0.19–6.29)	0.99(0.14–7.01)	0.54(0.21–1.37)	0.49(0.19–1.30)
LBW in singletons				
IPI < 6 mon	0.58(0.12–2.71)	0.75(0.13–4.41)	0.73(0.32–1.66)	0.70(0.30–1.65)
IPI 6–12 mon	Ref	Ref	Ref	Ref
IPI 12–24 mon	2.24(0.30–6.82)	3.15(0.31–4.45)	0.44(0.12–1.55)	0.41(0.11–1.51)
SGA in singletons				
IPI < 6 mon	1.25(0.28–5.45)	1.56(0.34–7.22)	0.95(0.31–2.88)	0.79(0.25–2.50)
IPI 6–12 mon	Ref	Ref	Ref	Ref
IPI 12–24 mon	2.24(0.30–6.82)	3.37(0.41–8.07)	2.02(0.66–6.16)	2.75(0.80–9.45)

*Abbreviations*: *OR* Odds ratio, *CI* Confidence interval, *IPI* Interpregnancy interval, *LBW* Low birth weight, *SGA* Small for gestational age

Adjusted for maternal age, body mass index, infertility duration, gravidity, parity, presence of tubal factor infertility, polycystic ovarian syndrome, endometriosis, diminished ovarian reserve, male factor or other infertility factors, the number of transferred frozen-thawed embryos, the developmental stage of transferred frozen-thawed embryos, the quality of transferred frozen-thawed embryos, the endometrial preparation protocols, endometrial thickness, and year of treatment

Regarding adverse neonatal outcomes, multivariable adjustment revealed that an IPI of < 6 months was not significantly associated with an increased risk of preterm birth (prior biochemical loss: aOR = 0.76, 95% CI 0.23–2.50; prior clinical loss: aOR = 0.71, 95% CI 0.35–1.43). Likewise, no significant differences were observed in the adjusted odds of LBW or SGA when comparing an IPI of < 6 months with an IPI of 6–12 months across both cohorts.

Sensitivity analyses for live birth were conducted after restricting the cohort to women younger than 35 years, cycles involving cleavage-stage embryo transfer, or cycles with good-quality embryo transfer (Supplementary Tables 1–3). Across all sensitivity analyses, no significant associations were observed between IPI length and live birth following subsequent FET, irrespective of the type of preceding pregnancy loss. In addition, subgroup analyses stratified by gestational age at clinical pregnancy loss and by abortion method among women with prior clinical pregnancy loss yielded consistent results (Supplementary Table 4), with no significant associations between IPI and live birth observed in any subgroup.

## Discussion

In this large retrospective cohort of women undergoing two consecutive FET cycles, we found that initiating a subsequent FET within six months after biochemical or clinical pregnancy loss was not associated with a reduced likelihood of clinical pregnancy or live birth, nor with an increased risk of adverse neonatal outcomes. These findings provide clinically relevant evidence that a short IPI following early pregnancy loss does not compromise subsequent ART outcomes and may not warrant routine postponement of treatment.

Our findings add important nuance to the existing and often conflicting literature regarding optimal IPI after pregnancy loss. Historically, recommendations to delay subsequent pregnancy attempts, most notably the World Health Organization guideline suggesting a minimum 6-month interval, were largely derived from observational studies in naturally conceiving populations, in which shorter IPIs were associated with increased risks of low birth weight, preterm birth, and premature rupture of membranes [8]. However, subsequent studies in natural conception settings have challenged this recommendation, demonstrating that shorter IPIs after pregnancy loss were not associated with increased adverse obstetric outcomes and, in some cases, were linked to improved live birth rates [10, 17, 18]. These discrepant findings may have been partly attributed to fecundity-related confounding, whereby women who conceive sooner inherently represent a more fertile subgroup [19].

In contrast, the ART setting offers a more controlled clinical environment, with standardized treatment timing, embryo characteristics, and luteal support, thereby substantially mitigating confounding related to underlying fecundity. Within this framework, our results suggest that the biological rationale for delaying pregnancy attempts after early loss may be less applicable to infertile women undergoing FET cycles, particularly when embryos are derived from prior stimulation cycles. Accordingly, counseling regarding treatment timing after loss may reasonably prioritize patient preferences and logistical considerations once medical readiness is confirmed.

Notably, our findings differ from those reported by Wang et al. [7], who observed inferior reproductive outcomes associated with shorter IPIs among women undergoing frozen blastocyst transfer after clinical pregnancy loss. These differences likely reflect important distinctions in study design and population. A critical distinction lies in the inclusion criteria; Wang et al. excluded biochemical pregnancy losses, focusing solely on clinical losses which may induce greater endometrial disruption and require a longer recovery. By including biochemical losses, our study reflects a broader spectrum of early pregnancy failure common in ART practice. Of note, even in our subgroup analysis of clinical pregnancy losses alone, we did not observe a detrimental effect of a shorter IPI, suggesting that endometrial recovery following early clinical miscarriage may be faster than previously hypothesized. Furthermore, because Wang et al. restricted their analysis exclusively to blastocyst-stage transfers, it is important to note that blastocyst transfer itself may be associated with an increased risk of certain adverse perinatal outcomes, including preterm birth and large-for-gestational-age infants [12, 13], which could confound associations attributed solely to IPI length. Importantly, we did not observe effect modification by embryo developmental stage, supporting the robustness of our findings across different transfer strategies.

Our results are also consistent with prior ART-based studies suggesting that short intervals after pregnancy loss do not necessarily impair subsequent outcomes. Sharon-Weiner et al. [20], in a smaller IVF cohort, reported no detrimental effect of shorter intervals between pregnancy loss and subsequent treatment on clinical pregnancy rates. Although limited by sample size and heterogeneous definitions of pregnancy loss, that study provides directionally concordant evidence, reinforcing the concept that routine postponement of ART following early pregnancy loss may lack biological justification.

Several biological and clinical considerations may further explain the absence of adverse outcomes associated with short IPIs in our cohort. In FET cycles, ovarian stimulation is avoided, endocrine perturbations are minimized, and embryos originate from oocytes retrieved in a prior cycle, thereby reducing the impact of progressive oocyte aging. In addition, endometrial preparation and luteal support are protocolized in ART treatment [21, 22], which may minimize variability in peri-implantation conditions across cycles. Importantly, our institutional protocol ensures that a subsequent transfer is only performed when the endometrium reaches a minimum thickness of 7 mm with an appropriate morphological pattern. This standardized clinical prerequisite guarantees a sufficient degree of endometrial recovery prior to embryo transfer, regardless of the precise calendar days elapsed since the pregnancy loss. Moreover, biochemical and early clinical pregnancy losses occur before substantial placental development or significant maternal nutritional depletion [7], which may explain the lack of association with adverse neonatal outcomes. Consistent with this interpretation, we observed no relationship between IPI length and preterm birth, low birth weight, or small-for-gestational-age infants.

The strengths of this study include its large sample size, consecutive FET cycles within the same individuals, standardized clinical protocols at a high-volume center, and detailed embryologic data, including embryo developmental stage. Comprehensive ascertainment of biochemical pregnancy loss further enhances the clinical relevance of our findings. Nevertheless, several limitations should be acknowledged. The retrospective, single-center design, along with the specific Chinese patient demographic and the relatively high proportion of cleavage-stage embryo transfers, limits causal inference and broader generalizability. Residual confounding from unmeasured factors, such as subtle variations in uterine recovery, psychosocial stress, or clinician-driven scheduling decisions, cannot be fully excluded. In addition, patients were not randomized to early versus delayed treatment, raising the possibility of selection bias. Furthermore, certain selection biases and methodological constraints inherent to our study design should be noted. First, by restricting the cohort to consecutive FET cycles from the same oocyte retrieval (to control for oocyte aging), we inherently excluded patients who conceived spontaneously after a loss or underwent fresh embryo transfers, which may limit the generalizability of our findings. Second, although our “< 6 months” category aligns with historical guidelines, the required clinical recovery protocols at our institution precluded immediate, next-cycle transfers. Consequently, our findings primarily establish the safety of reinitiating FET within a 2- to 6-month window, rather than immediately following a pregnancy loss. Future prospective, multicenter studies are warranted to confirm these findings and to refine evidence-based counseling regarding treatment timing after pregnancy loss.

In conclusion, among infertile women undergoing frozen–thawed embryo transfer following biochemical or clinical pregnancy loss, initiating subsequent treatment within six months was not associated with compromised reproductive or neonatal outcomes. These findings suggest that, in the absence of medical contraindications, individualized counseling, rather than routine treatment delay, may guide clinical practice, balancing biological safety with patient-centered considerations.

## Supplementary Information

Supplementary Material 1.

## Data Availability

The data are available upon reasonable request from the corresponding author.

## Abbreviations

IPI: Interpregnancy interval
FET: Frozen-thawed embryo transfer
aORs: Adjusted odds ratios
CI: Confidence intervals
ART: Assisted reproductive technology
WHO: World Health Organization
STROBE: Strengthening the Reporting of Observational Studies in Epidemiology
CMOH: Chinese Ministry of Health
HCG: Human chorionic gonadotropin
LBW: Low birth weight
SGA: Small for gestational age
SD: Standard deviation
