# Supplementary material for: Impact of interpregnancy interval after pregnancy loss on clinical pregnancy and neonatal outcomes of subsequent frozen-thawed embryo transfer cycles: a retrospective cohort study
Source: Reprod Biol Endocrinol. 2026 Apr 22;24:58. doi: 10.1186/s12958-026-01556-7 (PMC13235172; doi:10.1186/s12958-026-01556-7)
Supplement: Supplementary file 1 — Supplementary Material 1. [file 12958_2026_1556_MOESM1_ESM.docx]

Supplementary Table 1 Crude and adjusted odds ratios for live birth by interpregnancy interval in frozen embryo transfer cycles among women aged under 35 with prior pregnancy loss

|  | Number of cases (%) | Crude OR^a^ (95%CI) | Adjusted OR (95%CI) |
| --- | --- | --- | --- |
| Patients with biochemical pregnancy loss in preceding FET cycles | 1069 |  |  |
| IPI<6 month | 453/947(47.84) | 1.32(0.87–2.01) | 1.28(0.83–1.97) |
| IPI 6-12 month | 41/100(41.00) | Ref | Ref |
| IPI 12-24 month | 11/22(50.00) | 1.44(0.57–3.63) | 1.52(0.58–3.96) |
| Patients with clinical pregnancy loss in preceding FET cycles | 760 |  |  |
| IPI<6 month | 171/343(49.85) | 1.07(0.79–1.45) | 1.05(0.76–1.44) |
| IPI 6-12 month | 153/318(48.11) | Ref | Ref |
| IPI 12-24 month | 39/99(39.39) | 0.70(0.44–1.11) | 0.79(0.49–1.27) |

^a^Adjusted for body mass index, infertility duration, gravidity, parity, presence of tubal factor infertility, polycystic ovarian syndrome, endometriosis, diminished ovarian reserve, male factor or other infertility factors, the number of transferred frozen-thawed embryos, the developmental stage of transferred frozen-thawed embryos, the quality of transferred frozen-thawed embryos, the endometrial preparation protocols, endometrial thickness, and year of treatment.

Abbreviations: OR: odds ratio; CI: confidence interval; IPI: interpregnancy interval.

Supplementary Table 2 Crude and adjusted odds ratios of live birth in subsequent frozen-thawed cleavage-stage embryos transfer among patients with prior pregnancy loss by interpregnancy interval

|  | Number of cases (%) | Crude OR^a^ (95%CI) | Adjusted OR (95%CI) |
| --- | --- | --- | --- |
| Patients with biochemical pregnancy loss in preceding FET cycles | 1059 |  |  |
| IPI<6 month | 410/926(44.28) | 1.33(0.87–2.04) | 1.25(0.80–1.96) |
| IPI 6-12 month | 37/99(37.37) | Ref | Ref |
| IPI 12-24 month | 16/34(47.06) | 1.49(0.68–3.27) | 1.67(0.74–3.78) |
| Patients with clinical pregnancy loss in preceding FET cycles | 827 |  |  |
| IPI<6 month | 177/361(49.03) | 1.13(0.84–1.51) | 1.14(0.84–1.54) |
| IPI 6-12 month | 163/354(46.05) | Ref | Ref |
| IPI 12-24 month | 53/112(47.32) | 1.05(0.69–1.61) | 1.19(0.77–1.85) |

^a^Adjusted for maternal age, body mass index, infertility duration, gravidity, parity, presence of tubal factor infertility, polycystic ovarian syndrome, endometriosis, diminished ovarian reserve, male factor or other infertility factors, the number of transferred frozen-thawed embryos, the quality of transferred frozen-thawed embryos, the endometrial preparation protocols, endometrial thickness, and year of treatment.

Abbreviations: OR: odds ratio; CI: confidence interval; IPI: interpregnancy interval.

Supplementary Table 3 crude and adjusted odds ratios of live birth in subsequent frozen embryo transfer cycles with good-quality embryos among patients with prior pregnancy loss by interpregnancy interval

|  | Number of cases (%) | Crude OR^a^ (95%CI) | Adjusted OR (95%CI) |
| --- | --- | --- | --- |
| Patients with biochemical pregnancy loss in preceding FET cycles | 1179 |  |  |
| IPI<6 month | 482/1038(46.44) | 1.36(0.91–2.04) | 1.24(0.82–1.90) |
| IPI 6-12 month | 42/108(38.89) | Ref | Ref |
| IPI 12-24 month | 16/33(48.48) | 1.48(0.67–3.24) | 1.69(0.75–3.81) |
| Patients with clinical pregnancy loss in preceding FET cycles | 970 |  |  |
| IPI<6 month | 201/427(47.07) | 1.08(0.82–1.41) | 1.07(0.81–1.42) |
| IPI 6-12 month | 185/409(45.23) | Ref | Ref |
| IPI 12-24 month | 65/134(48.51) | 1.14(0.77–1.68) | 1.27(0.85–1.91) |

^a^Adjusted for maternal age, body mass index, infertility duration, gravidity, parity, presence of tubal factor infertility, polycystic ovarian syndrome, endometriosis, diminished ovarian reserve, male factor or other infertility factors, the number of transferred frozen-thawed embryos, the developmental stage of transferred frozen-thawed embryos, the endometrial preparation protocols, endometrial thickness, and year of treatment.

Abbreviations: OR: odds ratio; CI: confidence interval; IPI: interpregnancy interval.

Supplementary Table 4 Stratified analyses of the association between IPI and live birth in subsequent frozen embryo transfer among patients with a prior clinical pregnancy loss

|  |  | Number of cases (%) | Crude OR^a^ (95%CI) | Adjusted OR (95%CI) |
| --- | --- | --- | --- | --- |
| Trimester stage of preceding clinical pregnancy loss | Preceding clinical pregnancy loss in the first trimester | 856 |  |  |
|  | IPI<6 month | 207/439(47.15) | 0.96(0.72–1.28) | 1.01(0.74–1.36) |
|  | IPI 6-12 month | 159/330(48.18) | Ref | Ref |
|  | IPI 12-24 month | 46/87(52.87) | 1.21(0.75–1.94) | 1.46(0.88–2.42) |
|  | Preceding clinical pregnancy loss in the second trimester | 235 |  |  |
|  | IPI<6 month | 16/51(31.37) | 0.61(0.31–1.22) | 0.56(0.26–1.22) |
|  | IPI 6-12 month | 53/124(42.74) | Ref | Ref |
|  | IPI 12-24 month | 23/60(38.33) | 0.83(0.44–1.56) | 0.99(0.50–1.97) |
| Abortion method of preceding clinical pregnancy loss | Preceding clinical pregnancy loss with surgical evacuation | 566 |  |  |
|  | IPI<6 month | 136/304(44.74) | 0.84(0.59–1.20) | 0.90(0.61–1.32) |
|  | IPI 6-12 month | 99/202(49.01) | Ref | Ref |
|  | IPI 12-24 month | 29/60(48.33) | 0.97(0.55–1.73) | 1.02(0.55–1.89) |
|  | Preceding clinical pregnancy loss without surgical evacuation | 525 |  |  |
|  | IPI<6 month | 87/186(46.77) | 1.08(0.74–1.58) | 1.02(0.68–1.52) |
|  | IPI 6-12 month | 113/252(44.84) | Ref | Ref |
|  | IPI 12-24 month | 40/87(45.98) | 1.05(0.64–1.71) | 1.26(0.74–2.13) |

^a^Adjusted for maternal age, body mass index, infertility duration, gravidity, parity, presence of tubal factor infertility, polycystic ovarian syndrome, endometriosis, diminished ovarian reserve, male factor or other infertility factors, the number of transferred frozen-thawed embryos, the developmental stage of transferred frozen-thawed embryos, the endometrial preparation protocols, endometrial thickness, and year of treatment.

Abbreviations: OR: odds ratio; CI: confidence interval; IPI: interpregnancy interval.
